# Open-source CNC workstation for paper-based diagnostic assay assembly

**DOI:** 10.1016/j.ohx.2025.e00739

**Published:** 2025-12-30

**Authors:** Lucy Tecle, Shannon Riegle, Andrew Piepho, Jacqueline Linnes

**Affiliations:** a Purdue University, 610 Purdue Mall, West Lafayette, IN 47907, United States; b Indiana University School of Medicine, 340 W 10th St, Indianapolis, IN 46202, United States

**Keywords:** Computer numerical control (CNC), Open-source hardware, Automated fabrication, Low-cost instrumentation, Paper-based diagnostics

## Abstract

Paper-based diagnostics are promising for point-of-care testing, but their assembly is often manual and can introduce alignment variability. To address this, we developed a low-cost, open-source workstation that repurposes a three-axis computer numerical control (CNC) machine for automated pick-and-place assembly of paper-based assays. The system integrates an Arduino-based controller with GRBL firmware, a custom vacuum end effector, and component holders to handle delicate assay components. This design eliminates reliance on proprietary CNC controls, reducing the costs to under $900 while enabling machine-agnostic adaptability. Performance was validated across dipstick, lateral flow immunoassay, and custom duplex immunoassay formats. Linear placement accuracy averaged ∼0.5–0.6 mm (within functional tolerance for diagnostic readability), while angular deviations (1–3°) remained acceptable for sample flow. Control line intensity in CNC-assembled assays were statistically indistinguishable from hand-assembled assays, confirming preserved diagnostic performance. By lowering the barrier to automated fabrication, this workstation provides an accessible platform for academic labs, startups, and decentralized environments to prototype and scale paper-based diagnostics. The open-source hardware and design files expand opportunities for reproducible, affordable diagnostic assembly in early-stage research and development.

## Graphical abstract

**Figure:**
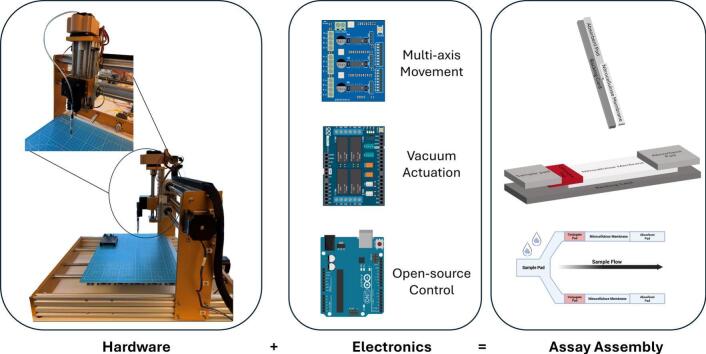

Specifications table.

**Table:** 

Hardware name	CNC-based Assay Assembly Workstation
Subject area	• Educational tools and open-source alternatives to existing infrastructure
Hardware type	• Biological sample handling and preparation
Closest commercial analog	Dobot Robotics MG400
Open-source license	All designs are released under the Creative Commons Attribution 4.0 International (CC BY 4.0) license.
Cost of hardware	$860 USD
Source file repository	https://doi.org/10.17632/89c957bzrg.1

## 1 Hardware in context

Point-of-care diagnostics are increasingly reshaping how health and safety challenges are addressed, offering rapid and decentralized testing. Among these, paper-based, microfluidic, and multiplexed diagnostic tools stand out for their affordability and adaptability across multiple industries such as medical devices, veterinary health, and food safety [1], [2], [3], [4], [5], [6]. Their reliance on low-cost materials and simplified fabrication makes them well-suited for point-of-need applications [7], [8], [9]. As demand for these devices grows [10], so does the need for scalable manufacturing. While corporations can rely on automated production pipelines, academic laboratories and start-ups face barriers in accessing manufacturing capabilities at a smaller and more affordable scale [11]. This gap inhibits clinical translation and underscores the need for fabrication approaches that are precise, cost-effective, and accessible to resource-limited developers.

Within this broader fabrication challenge, assembly is a commonly neglected stage of diagnostic development. Other aspects of assay fabrication, such as materials preparation, reagent-deposition methods, and even open-source reagents themselves are widely studied and accessible for lab settings [12], [13], [14], [15]. However, the physical integration of assay components in layers of thin films, lightweight adhesives, and porous paper membranes is often relegated to manual assembly or postponed until large-scale manufacturing. Since early-stage assay development is typically conducted in small batches with iterative prototype testing, it is not cost-effective to use commercial-grade equipment designed for high-volume production. The Dobot MG400 is the closest commercial analog in terms of function and benchtop scale, but its applicability to the handling or assembly of paper-based assay components has not been demonstrated in published work. As a result, manual assembly provides the closest practical and relevant benchmark for evaluating alignment and placement performance in this early-stage context. Hand-assembly of these fragile materials introduces misalignment, inconsistency, and handling errors, underscoring the need for automated pick-and-place workstations that can reliably transfer and align components. These issues undermine reproducibility and confound the optimization of critical design parameters during research and development [16], [17], [18]. Beyond immediate technical challenges, the absence of manufacturability considerations at this stage creates downstream barriers for scale up of designs that succeed in the lab but they are impractical or prohibitively expensive to scale. Addressing these challenges with an accessible, precise assembly system that can perform pick-and-place workflows would allow researchers to integrate considerations of alignment, reproducibility, and manufacturability much earlier in the R&D pipeline, ultimately bridging the gap between laboratory innovation and scalable production.

Automated systems have begun addressing the need for efficiency at scale within research environments. The use of robotic platforms has been seen in the biomedical sector with a promising impact towards operation time, error reduction, protocol modification, and quality of results [19], [20]. However, challenges arise with proprietary software, unique controllers, and limited customization options for small-scale R&D use [21], [22]. An open-source solution could better justify the integration of robotic handling for assay assembly in laboratory settings. Computer numerical control (CNC) machines are widely used in automation and precision manufacturing, offering an adaptable solution for diagnostic assay fabrication [23]. CNC machines typically run on G-code, an industry-standard language that translates commands into precise mechanical movements. Open-source software such as LaserGRBL and Lightburn has further expanded accessibility, enabling users to tailor CNC hardware to specific fabrication tasks [24]. Despite their accessibility, commercial CNC systems remain geared towards large-scale applications and often require outsourcing. By contrast, consumer-grade models are benchtop-friendly and affordable [25]. Typically designed for a single task such as engraving or cutting with optimal precision and performance, these smaller units operate at tunable speeds and forces that are suitable for handling the lightweight, fragile materials used in diagnostic assays. To be effective for assembly, however, more than one primary machining task would be required to support pick-and-place functionality. A customized workstation is necessary to balance functionality with the benefits of cost and accessibility.

A key modification for enabling pick-and-place functionality is vacuum-based handling. Conventional gripper end-effectors on CNC machines or robot arms provide secure, but not gentle, handling of target workpieces. Recently, soft robotic grippers have been developed; however, the thinness of most assay components does not make it a feasible option for the proposed application [26], [27]. In contrast, an end-effector with a vacuum-based tip can provide secure yet gentle placement of the delicate assay components. By integrating programmable motion with a vacuum nozzle, this system can apply sufficient force to adhere without damaging the assay. This design can also be customized for different assay formats through simple adjustments to end-effector height, nozzle size, or tubing configuration. In fact, modular micro-gripper formats can be easily integrated when open-source control of the CNC machine is possible, unlike its commercial counterparts [28]. With appropriate tuning, vacuum-based handling offers a low-cost, high-precision alternative to manual assembly, bridging the gap between early-stage research and scalable diagnostic production.

In this paper, we present an open-source CNC system for the automated assembly of paper-based diagnostic assays. The assembler consists of a 3-axis CNC frame equipped with a vacuum-powered nozzle as the end-effector with relay-controlled actuation, which is feasible through a microcontroller-based interface. We evaluated the system performance by measuring alignment precision and assembly efficiency across multiple assay formats, demonstrating applicability to diverse paper-based diagnostic designs. By lowering the barrier to automated fabrication, the system offers an accessible solution for academic laboratories, startups, and decentralized manufacturing environments, reducing reliance on manual methods and improving scalability of point-of-care diagnostics. This approach highlights how open-source, low-cost hardware can expand access to automated fabrication for early-stage diagnostic research.

## 2 Hardware description

The CNC-adapted assay assembler integrates a repurposed CNC framework, Arduino-based control electronics, and a custom vacuum end-effector. Unlike traditional CNC setups that rely on costly and proprietary controllers, this design leverages open-source GRBL firmware and Arduino hardware to enable automated pick-and-place assembly of paper-based assays at a fraction of the cost. The system’s modularity makes it adaptable across assay formats, while its low-cost and open-source architecture lowers barriers for research laboratories and startups seeking scalable diagnostic fabrication.

### 2.1 CNC-based framework

The system is built upon a three-axis CNC router machine (Vevor 3040 Engraver Milling Machine) repurposed for assay assembly. However, this design is machine-agnostic, as most CNC machines utilize GRBL firmware and operate through motorized multi-axis movement, providing high precision and repeatability, making them well-suited for applications beyond their traditional uses in engraving, milling, and robotic manipulation. In this design, open-source CNC control software such as LaserGRBL is used for movement programming. As for the stage, most CNC frames provide a similar working area suitable for assay formats. While a preassembled CNC machine’s frame and motors were initially used in this setup, the manufacturer’s costly controller box was not utilized to provide a lower-cost and open-source alternative. Most CNC engraving machines require a controller box for offline functionality. However, this project eliminates the need for a dedicated controller box, instead relying on a widely accessible microcontroller with modular shields custom electronic components to translate GRBL commands into precise linear movement, significantly reducing overall cost.

### 2.2 Electronics with control software

To replace the CNC’s proprietary controller, an Arduino UNO (Rev3) was selected and interfaced with two shields: a 4-channel relay shield and a CNC shield to control the vacuum pump and stepper motors, respectively. The Arduino UNO links the control software to the CNC hardware. The Arduino 4-relay shield switches the solenoid valve on or off as directed. The CNC shield, or grblShield (Syntheos, v5), provides a complete hardware solution for GRBL-based motion control [29]. Traditionally, an external G-code sender such as LaserGRBL is required to transmit commands from a computer to the Arduino UNO. However, by modifying open-source GRBL libraries, we enabled the Arduino to internally store and execute loops of G-code commands, thereby eliminating the need for external software senders. Selected for its three independent stepper motor drivers, the grblShield yields a centralized system working collectively on Arduino IDE open-source software. Custom scripts and libraries allow tailoring of movement and vacuum sequences, extending capabilities beyond standard CNC control. With appropriate customizations, the replacement of the CNC’s proprietary controller with an Arduino-based system enables scalable and reproducible automation of pick-and-place assembly for diagnostic fabrication. This adaptability provides an accessible alternative for laboratories and startups that require precise but cost-effective automation without investing in expensive commercial CNC controllers.

### 2.3 Vacuum mechanism

The barrel of a 1 mL syringe was repurposed as an end-effector to securely pick-and-place lightweight, delicate materials, such as the paper and plastic microfluidic assay components. The vacuum system consists of a DC power supply (24–30 V, 300 mA), solenoid valve, vacuum pump (1/4 Hp, 3.5 CFM), and tubing connection between the valve and syringe barrel. The syringe-based end-effector with a soft nozzle was developed to minimize damage to assay components.

• Low-cost, accessible automation: Replaces proprietary CNC controllers with open-source Arduino and GRBL components for affordable and adaptable applications
• Flexible, machine-agnostic framework: Compatible with different CNC platforms and adaptable for multiple assay formats
• Custom end-effector: Repurposed syringe-based, soft-tip vacuum mechanism enables precise pick-and-place handling for delicate paper substrates
• Scalable prototyping: Enables early-stage, iterative development of paper-based devices in both academic labs and resource-limited settings

## 3 Design files summary

Table 1 lists the design files required to reproduce the system and setup: (i) the sketch and library folder used in Arduino IDE for GRBL-based motion control, and (ii) STL files for 3D-printed component holders organized by assay format design. The control code was built upon an open-source GRBL repository [30]. This script assembles the LFIA design; however, this repository also has supplemental files for the duplex and dipstick assays.

**Table 1:** Design files for assembly and operation of the CNC workstation. Notations indicate the assay type where applicable (Dipstick^1^, LFIA^2^, Duplex^3^).

Design file name	File type	Open-source license	Location of the file
CNC_Arduino	.ino	MIT	https://doi.org/10.17632/89c957bzrg.1
User_Config	.h	MIT	https://doi.org/10.17632/89c957bzrg.1
CNC	.h	MIT	https://doi.org/10.17632/89c957bzrg.1
CNC	.cpp	MIT	https://doi.org/10.17632/89c957bzrg.1
Stage	.stl	CC by 4.0	https://doi.org/10.17632/89c957bzrg.1
Absorbent Pad Holder^1^	.stl	CC by 4.0	https://doi.org/10.17632/89c957bzrg.1
Nitrocellulose Membrane Holder^1,2^	.stl	CC by 4.0	https://doi.org/10.17632/89c957bzrg.1
Sample Pad Holder^1^	.stl	CC by 4.0	https://doi.org/10.17632/89c957bzrg.1
Conjugate Pad Holder^2^	.stl	CC by 4.0	https://doi.org/10.17632/89c957bzrg.1
Absorbent Pad Holder ^2^	.stl	CC by 4.0	https://doi.org/10.17632/89c957bzrg.1
Duplex Sample Pad Holder^3^	.stl	CC by 4.0	https://doi.org/10.17632/89c957bzrg.1
LFIA Holder^3^	.stl	CC by 4.0	hhttps://doi.org/10.17632/89c957bzrg.1

The Arduino sketch (CNC_Arduino.ino) implements GRBL-based motion control for the CNC workstation. [30]. Supporting files include *User_Config.h*, which defines component-specific setup parameters, and *CNC.h*, the header file for the CNC driver library. The corresponding implementation file, *CNC.cpp,* is a user library that handles UART communication between the Arduino and CNC via the grblShield, which depends on the ‘GRBL-Arduino-Library-master’ found in the official GitHub repository [30]. Users must install this library in their Arduino IDE before uploading the provided sketch folder. STL files are provided for 3D printing the component holders for each assay design.

## 4 Bill of materials summary

Table 2

**Table 2:** Bill of materials for CNC workstation. It includes components, specifications, costs, suppliers, and type for assembly and vacuum integration system. Items 1A and 1B are equivalent alternatives for the CNC router machine; the frame of 1A was used in this build, while 1B is a lower-cost option.

Designator	Component	Key Specifications	Quantity	Unit Cost − USD	Total Cost − USD	Source of Materials	Type
1A	CNC Router Machine	3040 Engraver Milling, Offline Controller, Limit Switches, E-stop, 3 Axes, 400 x 300 x 100 mm)	1	660	660	Vevor	Framework
1B	CNC Router Machine	60 W, 3 Axis GRBL Control, 300 x 200 x 60 mm	1	253	253	Vevor	Framework
2	Uno Rev3	N/A	1	27.6	27.6	Arduino	Electronics
3	4 Relays Shield	N/A	1	27.6	27.6	Arduino	Electronics
4	grblShield	gshield V5	1	63	63	Synthetos	Electronics
5	Panasonic Capacitor	47 µF	1	0.38	0.38	DigiKey	Electronics
6	Regulated DC Power Supply	1671A, 0–30 V DC, 0–3 A, linear, single output	1	350	350	BK Precision	Electronics
7	ASCO Solenoid Valve	N/A	1	71	71	Furnace Part Source	Hardware
8	Vacuum Pump	1/4Hp, 3.5 CFM	1	60	60	Vevor	Hardware
9	Disposable Syringes	1 mL, Leur-lok tip	1	1.03	1.03	BD	Hardware
10	Plastic Dispensing Needles	14 G, 1/2″ PP blunt Luer lock tip, flexible needle	1	0.35	0.35	Amazon	Hardware
11	Tubing (vacuum pump-valve)	1/4 in. I.D. x 3/8 in. O.D. x 10 ft. Clear Vinyl	1	3.45	3.45	Home Depot	Hardware
12	Tubing (valve-syringe)	Masterkleer PVC Plastic 5233 K51 (1/8″×1/16″)	2	0.58	1.16	McMaster-Carr	Hardware
13	Reducer (tubing connection)	USA Sealing Push-to-Connect Tube Fitting	1	3.16	3.16	Motion	Hardware

## 5 Build instructions

### 5.1 Assembly of CNC-based workstation

**Fig. 1:**
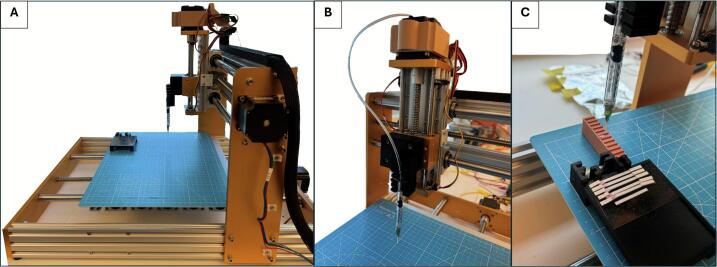
Overview of the hardware system for automated component pick-and-placement. (A) Full view of the CNC-based 3-axis frame used for motorized positioning during assay assembly. (B) Close-up of the Z-axis-mounted end-effector, a modified 1 mL syringe connected to an external air pump for vacuum-based pick-and-place. (C) Close-up of the placement stage with custom 3D-printed holders for assay components.

The 3-axis CNC frame consists of the working stage and robotic arm that is powered by 3 stepper motors, as shown in Fig. 1. Each stepper motor represents one of the 3 axis directions (X/Y/Z) and wires from each direction are connected to the gshield outlined later in this section. Similarly, each motor has limit switches in each +/-direction with electrical leads connected to the gshield. They are adhered to the CNC frame and triggered by the stage when moving. The robotic arm is supplemented by the syringe-based end effector and camera mount, which is also outlined in this section. A grid-lined cutting board mat is adhered to the working stage and 3D printed holders were adhered to the mat with stacked pre-cut to size paper components of the assay. All components were stacked facing upward. Their home positions and number of components will be needed for the main script outlined in this section.

### 5.2 Electronics

**Fig. 2:**
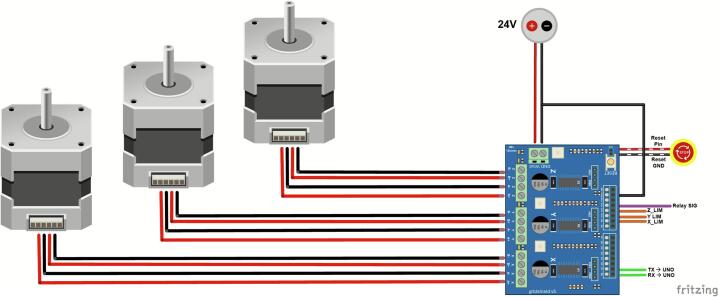
Pinout diagram demonstrating the electronic setup for motorized control of the CNC-based system. A GRBL-compatible shield interfaces with three stepper motors to drive the X/Y/Z axes for precise and repeatable positioning. Limit switches are connected to pins D9-11 to enable homing functionality for each axis. Pin D12 is configured to synchronize vacuum actuation with CNC movement via the 4 Relays shield.

The wiring diagram can be seen in Fig. 2. The Arduino UNO, relay shield, and gshield boards are stacked upon each other from bottom to top, respectively. A simplified pinout map for this configuration is provided in Supplemental Fig. S1 to highlight essential logical connections. Connectors are needed between the relay shield and gshield. The Arduino UNO is powered through the USB port and connected to a computer with the source code. The Arduino 4 Relays board drives high power loads to control the vacuum. The common (C) and normally open (NO) terminals were connected to DC 24 V and ground, respectively. To avoid shorting, an electrolytic capacitor was added between the DC input and C terminal. Pin 12 of the relay shield controls Relay 4 to enable ON/OFF functions for the vacuum. The CNC gshield connects the three stepper motors to each axis of the CNC device’s movement. Pins D9, D10, and D11 were configured for limit switches for the X/Y/Z direction, and pins D0/D1 provide UART communication between the UNO and gshield. The pinout diagrams of the relay and gshield should be referenced to ensure no competing or limiting functions. For example, Relays 1, 2, and 3 are controlled by pins 4, 7, and 8; however, these same 3 pins on the gshield are designed for controlling the stepper motors and not general-purpose digital switching pins. Hence, it is important to ensure proper power supply voltage, check for loose connections, and verify the relay assigned for vacuum control.

### 5.3 Vacuum integration with syringe end-effector

**Fig. 3:**
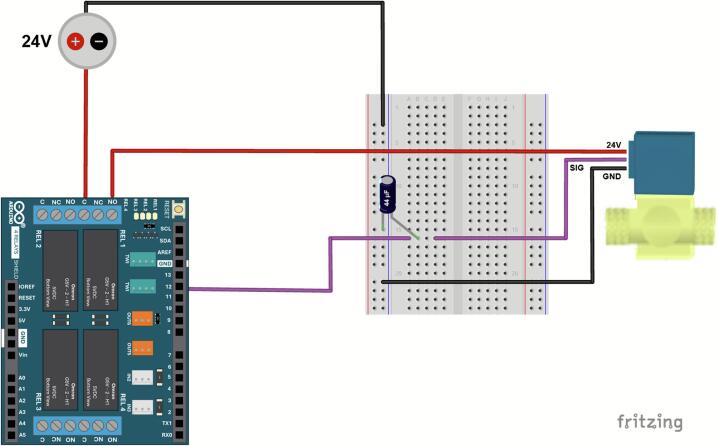
Pinout diagram of the electronic setup for valve actuation in the vacuum system. An Arduino 4 Relays shield controls a solenoid valve connected to the vacuum pump through Relay 4 via pin D12 for the input signal. A 44µF capacitor is added to provide electrical isolation and protect against voltage spikes during high-power switching.

In Fig. 3, a picture of the setup is shown. A vacuum pump was attached to one end of the tubing and the other end leads to a solenoid valve. The valve was wired to ground, the signal produced by the relay shield, and tubing to the CNC device’s end effector. A DC power supply box provides 24–30 V to operate the valve and a current output of 300 mA is expected. Before complete integration into the assembly process, confirm the vacuum pressure is sufficient and operating correctly. A constant pressure pump was used here, but a digital pump can be used for adjustable suction strength. For the end-effector, remove the plunger of a 1 mL syringe and insert the tubing that is connected to the vacuum’s stepper motor. In lieu of a needle, a silicone tip with a luer lock was used for an air-tight seal. A 3D printed holder was designed to securely hold this custom end-effector, as seen in black within Fig. 1B, with additional grooves to manually adjust its height when used for different projects.

### 5.4 Custom CNC-Arduino script/library for assay assembly

#### 5.4.1 Software architecture

The Arduino program is structured with a main (.ino) file that manages the control logic, calling functions defined in CNC.cpp and declared in CNC.h and User_Config.h. The CNC.h file declares function prototypes and includes necessary dependencies, while the CNC.cpp file implements these functions and contains the logic for CNC motion, homing, and vacuum control. Notably, the CNC.cpp utilizes GRBL commands to send movement instructions. The main script calls upon high-level functions, such as initializeCNC(), absoluteMove(), and relativeMove() to control the CNC system.

#### 5.4.2 User-defined inputs

All user-tunable parameters (coordinates, offsets, delays, and run size) are centralized in User_Config.h. Common toggles to run various assay formats are exposed as Boolean flags. If new or additional components are introduced, the user can duplicate or substitute an existing parameter and its call function in the.ino file. This enables users to primarily edit a single file to adapt the system to alternative assay layouts. Table 3 categorizes the variables based on first-time setup, project-specific, and routine use. These inputs govern critical functions such as defining component positions, adjusting for height changes as parts are picked up, and ensuring safe movement paths. Maintaining accurate input values is essential to prevent misalignment and ensure consistent operation.

**Table 3:** User-defined CNC parameters grouped by setup stage, project-specific adjustments, and routine operation within *User_Config.h*. The terms ‘part’ and ‘component’ are interchangeable. Text in parentheses will vary based on selected part and axis.

Category	Parameter(s)	Description
**First-Time Setup**	baseHeight, safeHeight	Maximum and minimum resting Z-positions
	placementArea_(x/y/z)	Reference coordinates for placement stage

**Project-Specific**	(part)Holder_(x/y/z)	Center coordinates of each component in holders
	assay_x_placement_offset	Lateral placement offset between successive assays
	(part)_y_placement	Y-axis placement position of each component per assay
	(part)_z_placement	Contact height position of each component at placement
	(part)_z_pickup_offset	Per-assay height offset for materials in holders

**Routine Use**	numAssays	Defines the number of assays to assemble per run
	run_(part)	Toggle which components to assemble per run

#### 5.4.3 Homing function

A homing function enables the CNC to establish its position in real space at the start of each run. Upon upload, each axis advances in 1 mm increments towards its limit switch and then retracts 10 mm to set a repeatable zero position. Reducing the step size improves positioning precision but extends the cycle time as each axis is homed sequentially. In our setup, the homing cycle takes ∼ 90 s. The GRBL firmware prevents assembly motion until the homing cycle is complete. This process yields a potential variance of up to 1 mm per axis per run. Currently, the 1 mm resolution is considered acceptable, but this can be tuned as needed to balance accuracy against cycle duration.

#### 5.4.4 Error handling

To ensure reliable operation, the initializeCNC() function resets the system at startup. It clears buffers, resets GRBL execution states, and performs a soft reset to prevent motion errors. If homing is enabled, it places the system into an alarm state until homing is completed, ensuring all movement commands execute from a known reference position. For additional safety, an emergency stop button can be connected to the CNC gshield’s Reset Pin (J12 + ) and GND Pin (J12-). This allows immediate halting of CNC operations if needed, preventing potential damage or misalignment.

#### 5.4.5 Calibration Procedures

Calibration is required to ensure accurate component pickup and placement. These steps should be completed after the holders are mounted to the assembly stage and updated per assay design changes. In the following steps, the user is instructed to send G-code commands for ‘jogging’ (step-wise movement) of the end-effector. To jog, use the Arduino IDE serial monitor or a free G-code sender software. If using the latter option, ensure the appropriate USB-to-serial driver is installed (e.g. CH340/341). G-code syntax is officially defined in ISO 6983–1:2009 [31]; for axis indexing, $G1 provides linear movement commands to specified endpoints defined by the X/Y/Z address. See Table S1 in the Supplementary Information for common CNC/GRBL commands to assist with calibration. The instructions below include italicized parameters that correspond to Table 3 and should be updated in *User_Config.h*.

(A) Define safety heights
  a. Jog the end-effector to determine the tallest or highest point it can safely traverse. Set this Z-position as *safeHeight*.
  b. Jog the end-effector to determine the shortest or lowest point it can safely traverse. Consider the holders’ height to avoid collisions during assembly and idle state. Set this Z-position as *baseHeight*.
(B) Define placement reference
  a. Position the adhesive backing card (sticky side up) to the stage.
  b. Identify a reference corner on the stage where first assay will be located.
  c. Jog the end-effector to the selected corner. Record and set these coordinates as *placementArea_(x/y/z)*. All components will be placed based on offsets from this reference point.
(C) Calibrate holders
  a. Mount the 3D-printed holders along the collection zone at x  = 0.
  b. Load components into holder. Ensure consistent count for each run.
  c. Jog the end-effector to the geometric center of the first component’s holder. Record the X/Y coordinates for *(part)Holder_(x/y)*.
  d. Jog the end-effector to lower it until it is 1–2 mm above the component but not directly touching. Record the Z value for *(part)Holder_z*.
  e. Repeat for each component’s holder.
(D) Define placement location and pickup offsets
  a. Place an assembled assay on the stage along the x-axis as a placeholder.
  b. Jog the end-effector to the first component’s target location. This should be at the center of the component with light contact. Record the X position for *assay_x_placement* and the Y/Z positions for *(part)_(y/z)_placement* parameters.
  c. Repeat and record the Y/Z positions for each component.
  d. Using a caliper or product spec sheet, measure each component’s thickness (in mm). Set this value for *(part)_z_pickup_offset*.
  e. (Optional) By default, holders are vertically stacked. For components prone to static adhesion (e.g. glass fiber), arrange them in a row using a horizontal (side-by-side) holder to measure the distance (in mm) between each component’s center. Set this value as the *(part)_x_pickup_offset*.
(E) Validate and optimize calibration
  a. Set *numAssays* and *run(part)* to 2 and true, respectively.
  b. Verify that the sketch compiles without errors. Common mistakes include missing libraries and mismatched parameter names.
  c. Switch on the vacuum and run the sketch to monitor assembly for components missed during pickup or incorrectly placed upon stage. Reconfigure parameters for optimal assembly.
  d. (Optional) To adjust delay times, update *t_(1/2/3/4)* in *User_Config.h* per assembly step or component as needed.

## 6 Operation instructions

1. Place each component stack in its 3D-printed holder along the collection zone (x = 0).
2. Place an adhesive backing card on the placement/stage area.
3. Set the DC power supply box to 24 V and turn it on.
4. Switch on the vacuum pump.
5. Plug the controller into the computer via USB.
6. Open the sketch folder (CNC_Arduino.ino, User_Config.h, CNC.h, CNC.cpp) in Arduino IDE. Ensure the GRBL-Arduino-Library-master is installed in the Arduino libraries folder (see *Design Files Summary*).
7. Set run parameters (refer to Table 2 and Calibration). Confirm that the components and coordinates correspond to the holders’ locations.
8. Click Upload for homing cycle to begin (∼60–90 s, varies based on location from last use)
9. Monitor during assembly for external obstructions or complications. If needed, use the E-stop switch.
10. Remove assembled strips from stage (and cut backing card to individual assay size).
11. Power down vacuum pump and DC supply.

## 7 Validation and characterization

Several assay formats were evaluated using the CNC workstation for their varied complexity. Dipstick assays were comprised of 2 components and lateral flow immunoassay (LFIA) were assembled with 4 components. The duplex assay used two commercially available LFIA (USTAR Biotechnologies) modified without sample pads and substituted with an in-house customized sample pad for the duplexed assay format. The new sample pads of the duplex and conjugate pad for the LFIA were made of glass fiber, requiring more delicate handling than the thicker, more robust cellulose or cotton pads used for other assay components. All material specifications and component dimensions can be found in Table S2. There are two primary motives for these studies: 1) assess the precision of assembling multiple components and 2) whether automated assembly would affect the assay results.

Dimensional tolerances for CNC-based assay assembly were informed by machining standards and diagnostic device specifications. ISO 2768–1:1989 provides general-purpose tolerances for assembled parts, specifying ± 0.3 mm and ± 0.5 mm for linear dimensions in the 30–120 mm and 120–400 mm range, respectively [32]. In the context of LFIA assembly, cassette design patents indicate that placement tolerances up to ± 0.5 mm can be accommodated without impairing strip readability or function [33]. These defaults align with requirements where pad overlap and line visibility define functional limits**.** As for angular displacement, it was not defined in cassette patents and has no equivalent device-specific benchmark. In this work, general-purpose angular tolerances in ISO 2768–1 (±0.2-3°) are treated as conservative defaults and acknowledged in Appendix A to not reflect functional performance when these limits are exceeded [32]. ISO 22081:2021 reinforces defining tolerances by functional need [34]. This aligns with ISO 13845:2016, which requires medical device manufacturers to establish functional tolerances within quality management systems, where no universal angular benchmark is defined [35].

Functional acceptability was therefore based on observed diagnostic performance at measured component interfaces, particularly where angular deviations influence sample transfer toward the detection membrane. Based on these combined considerations, ±0.5 mm was adopted as a practical benchmark for linear placement, while angular tolerance was treated as a functional parameter defined by diagnostic performance.

Linear and angular displacements of individual components were assessed from scans of assembled assays using ImageJ (Fiji) and custom Python scripts developed for this study. All linear measurements were scaled to convert from pixels to millimeters. For individual pad placement, linear displacement was measured as the x-axis offset (in mm) between adjacent assay components (e.g. sample pad-conjugate pad). Positive and negative values represented leftward and rightward shifts, respectively. Angular displacement was calculated as the rotation (in degrees) of each component relative to the deviation from the absolute vertical orientation (90°). Using the angle tool in ImageJ, this was determined by tracing a reference line along the width of the assay and creating an angle along the x-axis edge of the assay component. The deviation from an absolute right angle was reported as a positive (clockwise) and negative (counterclockwise) value. All measurements were performed manually, which may introduce operator variability and pixel-level error; however, this approach was appropriate for functional tolerance ranges.

Statistical analyses were performed in Python (SciPy). One-sided t-tests were used to determine whether linear and angular displacements differed significantly from the ideal alignment. Welch’s t-tests were applied to compare control line intensities between manual and CNC assemblies. Levene’s tests were used to evaluate differences in variance, providing a measure of reproducibility across assay components. A significant threshold of p < 0.05 was applied for all analyses.

**Fig. 4:**
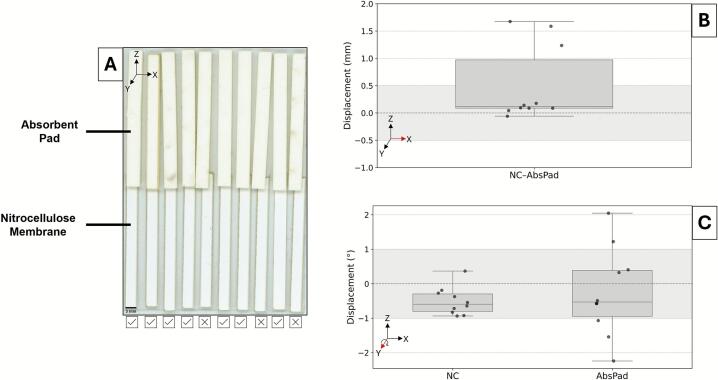
Component placement accuracy in dipstick assembly by the CNC system. (A) Scan of the nitrocellulose membrane (NC) and absorbent pad (AbsPad) interface across 10 dipsticks, with checkmarks and ‘X’ marks denoting assemblies within or outside the ± 0.5 mm tolerance, respectively. (B) Vertical displacement of the NC-AbsPad overlap. Shaded region denotes the target ± 0.5 mm tolerance. (C) Angular displacement of NC and AbsPad components. Shaded region denotes the target ± 1° tolerance. Axis references indicate assembly and displacement orientation.

Fig. 4 visualized and quantified the CNC-assembled dipstick assays. Across 10 assays, the nitrocellulose and absorbent pad interface exhibited an average magnitude of vertical displacement of 0.52 ± 0.69 mm, where 70 % of assays were within the ± 0.5 mm specification. The average magnitude of angular misalignment was 0.58 ± 0.27° and 1.05 ± 0.71° for nitrocellulose and absorbent pad, respectively. In addition to larger mean angular offsets, the absorbent pad exhibited significantly higher variability than the nitrocellulose membrane (Levene’s test, p = 0.034), indicating reduced reproducibility in placement across assays. While the nitrocellulose components were within range, half of the absorbent pad were not. Variability was likely due to minor shifting within the absorbent pads’ holders. It may also have been influenced by the use of a singular head for the end-effector, which could permit greater rotation compared to a dual-headed configuration for multiple contact points. A tighter fitting holder or tunable pressure control could address an unstable hold of this relatively heavier component. Considering samples for dipstick assays have first contact with the nitrocellulose and the elongated absorbent pad allows for adequate wicking, these CNC-assembled assays are expected to support reproducible capillary flow and result interpretation.

**Fig. 5:**
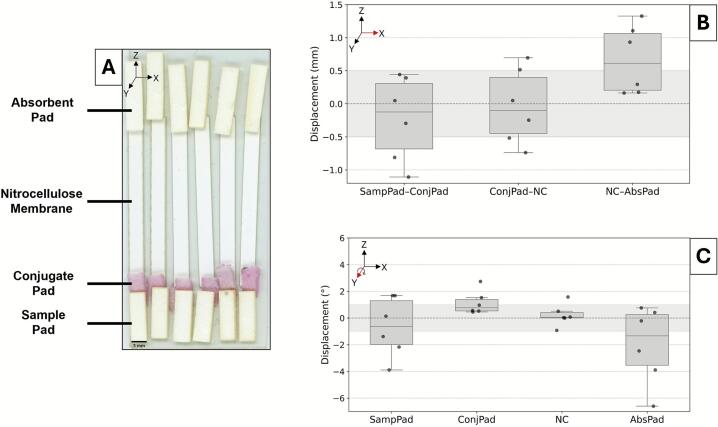
Component placement accuracy in LFIA assembly by the CNC system. (A) Scan of assembled LFIA strips labeled with the sample pad (SampPad), conjugate pad (ConjPad), nitrocellulose membrane (NC), and absorbent pad (AbsPad). (B) Vertical displacement (mm) of LFIA component interfaces. Shaded region denotes the target ± 0.5 mm tolerance. (C) Angular displacement (°) of individual LFIA components. Shaded region denotes the target ± 1° tolerance. Axis references indicate assembly and displacement orientation.

In Fig. 5, the vertical and angular displacement for LFIA assembly were quantified. The average magnitude of vertical displacement was 0.52 ± 0.38 mm, 0.46 ± 0.27 mm, and 0.67 ± 0.52 mm for sample pad to conjugate pad, conjugate pad to nitrocellulose, and nitrocellulose to absorbent pad, respectively. The nitrocellulose to absorbent pad interface primarily fell outside of acceptable tolerance range, whereas the other two overlaps averaged within or very close to this benchmark. The average magnitude of angular displacement was 1.86 ± 1.20°, 1.13 ± 0.89°, 0.66 ± 0.60°, and 2.39 ± 2.50° for the sample pad, conjugate pad, nitrocellulose, and absorbent pad, respectively. Although angular deviations were largest at the absorbent pad, variance was not significantly different across components (Levene’s test, p = 0.073), suggesting comparable reproducibility despite the larger offsets. Unlike the dipstick assay, the absorbent pad for the LFIA is approximately half the length yet doubled in angular displacement. The sample pad was comprised of similar material as the absorbent pad, drawing similarities in their wider distribution of misalignment compared to the conjugate pad and nitrocellulose components. This repeated variability reinforces the need for tunable pressure. Overall, CNC assembly of LFIAs can still be considered functionally successful, as the reliable placement of nitrocellulose membranes remains visible through the cassette window. Minor angular shifts of the sample pad and conjugate pad are unlikely to affect performance because most commercial cassettes incorporate grooves and alignment pins that secure and straighten the strip [33]. Hence, CNC assembly can be deemed suitable for multi-component, paper-based assay formats.

**Fig. 6:**
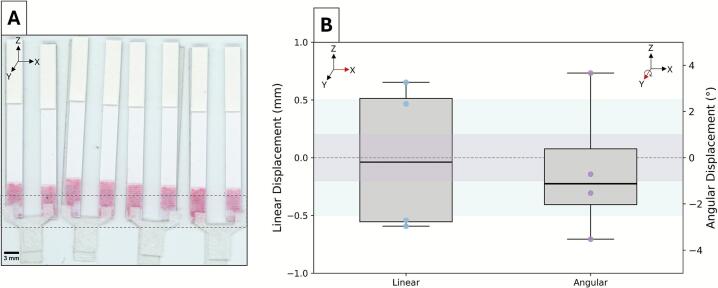
Linear and angular displacement per CNC-assembled duplex assay. (A) Scan of assembled duplex assays with a dotted box indicating the region of interest at the sample pad and conjugate pad interface. (B) Linear (mm, blue) and angular (°, purple) displacement measured for each duplex assay. (For interpretation of the references to colour in this figure legend, the reader is referred to the web version of this article.)

For the CNC-assembled duplex assays, the interface between the glass fiber sample pad and the two conjugate pads of the commercial LFIAs was evaluated. In the assembly sequence, the two LFIAs were first positioned on the backing card before the sample pad was placed to overlap both conjugate pads. The average magnitude of linear and angular displacements was 0.56 ± 0.08 mm and 2.36 ± 1.47°, respectively (Fig. 6). While the linear displacement was marginally above the 0.5 mm threshold, the wider conjugate pads accommodated these minor offsets. Angular displacement significantly varied, likely due to the fragile, compressible nature of glass fiber and challenges in consistently engaging the pad at its center with the vacuum-based end effector. Prior work on suction- and adhesion-based grippers has shown that reliable handling strongly depends on object geometry and the center-point engagement, as off-center pickup introduces torque and reduces seal stability [36], [37]. In contrast, the relatively heavier pre-assembled LFIA strips were positioned more consistently, suggesting that the uniform end-effector pressure was well tolerated by robust components but less forgiving for lightweight, delicate materials. Overall, these results demonstrate that the CNC system maintained placement precision close to the practical tolerance limits, even under more versatile material handling of duplex assay assembly.

**Fig. 7:**
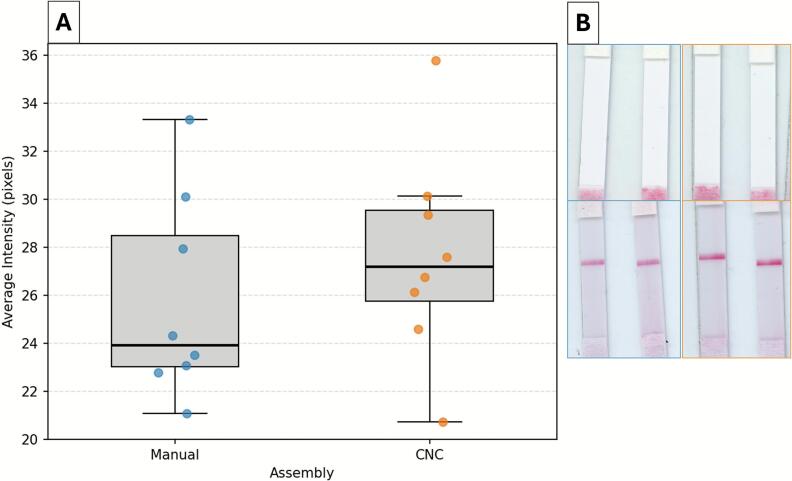
Control line intensity for manual versus CNC-assembled duplex assays. (A) Quantification of the average control line pixel intensity for manual and CNC-assembled duplex assays. (B) Representative scans of nitrocellulose membranes before (top row) and after (bottom row) sample addition for duplex assays assembled by hand (blue outline) and by CNC (orange outline). (For interpretation of the references to colour in this figure legend, the reader is referred to the web version of this article.)

To evaluate whether the automated assembly compromised assay performance, the assay control line intensity was compared between manual and CNC-assembled duplex assays (Fig. 7). While the CNC-assembled assay pixel intensity was slightly stronger (27.63 ± 4.4) than the manual assembled assays (25.77 ± 4.2), the groups were statistically indistinguishable (Welch’s *t*-test, p > 0.05). These comparable results indicate that CNC assembly maintains assay functionality relative to manual assembly. They also suggest that the positional accuracy achieved by CNC assembly is sufficient to ensure consistent reagent interaction and flow, even in the more complex duplex format**.** Excluding its outlier, the distribution of CNC-assembled duplex assays exhibited a slightly tighter dispersion, reflecting internal consistency within the automated workflow. CNC assembly also reduced build time by approximately two minutes for the duplex assay (manual: ∼5 min; CNC: ∼3 min, n = 4), indicating a potential throughput advantage. Future investigations are needed to assess inter-operator variability for broader reproducibility comparisons. Collectively, these findings support CNC assembly as a viable alternative to manual fabrication without loss of assay sensitivity, particularly when combined with a silicone-tipped end effector to minimize the risk of membrane damage.

While these studies demonstrate the feasibility and accuracy of CNC-based assembly for paper diagnostics, several limitations remain that present opportunities for future improvements. The current system relies on a fixed-pressure pneumatic setup, limiting its ability to adjust suction for components of different mass or fragility. A potential improvement would be to configure the unused relay channels so they actuate additional vacuum branches, each with a different fixed flow restriction to provide discrete vacuum levels based on the material type. Implementing this redesign would require additional valves and tubing for each branch. It will also necessitate verification that multi-relay operation remains within safe power limits and does not interfere with communication across the current build’s stacked board configuration. Beyond vacuum control, the single-head end-effector was effective for lightweight materials but exhibited greater variability with heavier absorbent pads, suggesting that multi-point contact or a larger-diameter end-effector could improve placement consistency. A dual-head suction cup may improve handling stability during placement, or the aforementioned approaches for tunable pressure control could mitigate this handling limitation without changing the end-effector design. Next, manual loading of components introduced user-dependent variability and occasional static adhesion; these effects could be mitigated through anti-static pretreatment or by adopting a horizontal loading configuration at the expense of occupying more stage area with component holders.

Reproducibility considerations extend beyond hardware constraints, particularly when comparing automated assembly to manual methods. Comprehensive reproducibility comparisons remain outside the scope of this work as meaningful assessment would require accounting for operator skill level, assay complexity, and task-specific performance criteria. In addition, the current workstation evaluates alignment through post-assembly image analysis. Integrating real-time visual feedback could reduce placement errors, but this add-on remains beyond the scope of this work due to challenges in software integration for remote camera control and the need for high image resolution to detect millimeter-scale misalignment. Finally, the selection of our specific grblShield may raise concerns about long-term accessibility as newer models enter the market. However, because the Arduino Uno runs standard GRBL firmware uploaded through the Arduino IDE, the control logic itself is not dependent on this particular board. Future iterations can adopt alternative shields, requiring only pinout remapping to accommodate differences in how step, direction, and limit-switch signals are routed. This flexibility ensures that the workstation can be readily adapted to new hardware as the open-source CNC ecosystem evolves. Collectively, these considerations outline clear opportunities for improving reproducibility, handling robustness, and material compatibility in future iterations/applications of the workstation.

With these areas for future improvements in mind, the results demonstrate that the CNC workstation can reliably assemble diverse assay formats with placement accuracy. Across all three assay formats, functional performance remained consistent at the measured angular deviations, supporting that the 1-3° range observed remained within functional limits for sample transfer and fluid flow. Together, the combination of consistent linear placement, acceptable angular performance, and preserved diagnostic signal quality supports the system’s suitability for prototype fabrication and benchtop-scale assay development.

### 7.1 Validation summary

• Positional accuracy: CNC assembly achieved linear placement within ∼ 0.5–0.6 mm for all assay formats, nearly within the predefined tolerance limit for multi-component assay formats.
• Angular tolerance: Angular deviations were typically 2-3°, exceeding the 1-2° ISO tolerance threshold but remaining within functional limits for assay fluid continuity. Angular shifts were most pronounced at the membrane-absorbent pad interface, but cassette alignment features mitigate their impact.
• Component handling: A silicone-tipped end-effector minimized membrane damage, and tunable pressure control may further improve fragility across substrates.
• Reproducibility and robustness: Variability in placement was comparable across dipstick, LFIA, and duplex assemblies, with no single format exhibiting significant misalignment.
• Diagnostic readout: CNC assembly preserved control line intensity, validating that minor positional deviations did not affect interpretation.

## Ethics statements

This work did not involve human participants or animal experiments.

## CRediT authorship contribution statement

**Lucy Tecle:** Writing – original draft, Visualization, Methodology, Investigation, Formal analysis, Data curation. **Shannon Riegle:** Writing – review & editing, Validation, Software. **Andrew Piepho:** Investigation, Data curation. **Jacqueline Linnes:** Writing – review & editing, Supervision, Funding acquisition, Conceptualization.

## Declaration of competing interest

The authors declare the following financial interests/personal relationships which may be considered as potential competing interests: Jacqueline C. Linnes is co-founder of EverTrue LLC, a diagnostics company developing paper-based POC tests. All other authors have declared that they have no competing interests.
