# Supplementary material for: Open-source CNC workstation for paper-based diagnostic assay assembly
Source: HardwareX. 2025 Dec 30;25:e00739. doi: 10.1016/j.ohx.2025.e00739 (PMC12808598; doi:10.1016/j.ohx.2025.e00739)
Supplement: Supplementary Data 1 [file mmc1.docx]

**Supplementary Material**


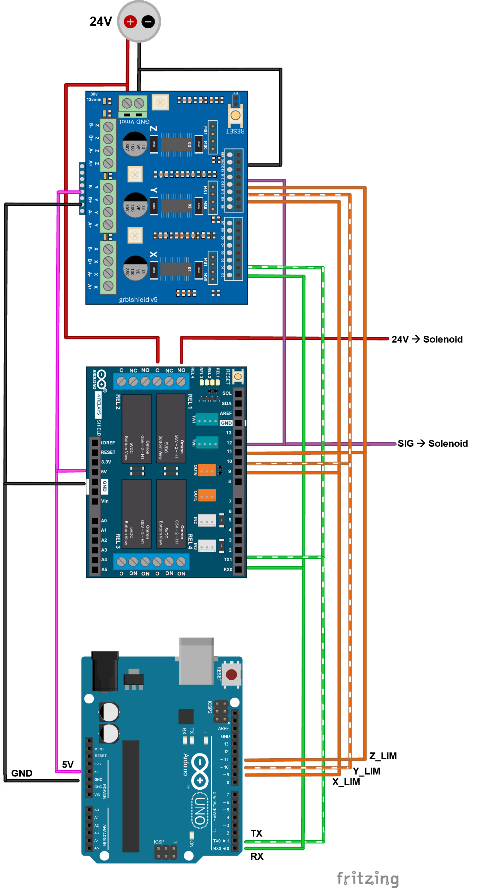


Figure S1: Pinout diagram showing logical connections between the Arduino UNO, 4-Relay shield, and gshield. Pins D0 and D1 provide UART communication, pins D9-11 are configured as limit swtich inputs for the X/Y/Z axes, and pin D12 serves as the relay control signal. The UNO supplies 5V, the external power supply box provides 24V, and both yield common grounds. Color coding: red = 24V, black = GND, pink = 5V purple = relay signal, orange = limit switch, green = RX/TX. Banded wires are for visual clarity.

Table S1: Common CNC/GRBL commands for initialization. Commands can be executed using third-party GRBL control software or Arduino IDE serial monitor.

| **Common CNC/GRBL Commands for Calibration** | | |
| --- | --- | --- |
| **Command** | **Function** | **Example** |
| G90/G91 | Set absolute/relative positioning | G90, G91 |
| G0/G1 | Define linear movement to specified endpoint | G1 X5Y5Z20 |
| G4 P# | Enables dwell for # seconds | G4 P20 (20s hold) |
| F# | Set feed rate (mm/min) | G1 X5Y5Z20 F2000 |
| M3/M5 | Enable/disable spindle spinning | M3 (toggle on)  M5 (toggle off) |
| S# | Set spindle speed (RPM); adapted for solenoid valve toggle | S0 (vacuum on)  S1000 (vacuum off) |

Table S2: Assay Components used for Dipstick, lateral flow immunoassay (LFIA), and Duplex assembly. This includes material specifications and dimensions.

| **Component** | **Product Name** | **Manufacturer** | **Catalog No.** | **Dimensions**  **(LxW, mm)** | **Application** |
| --- | --- | --- | --- | --- | --- |
| Nitrocellulose membrane | Whatman FF120HP | Cytiva | 13549205 | 30x3 | All |
| Conjugate pad | Whatman Standard 14 | Cytiva | 8133-2250 | 5x3 | LFIA |
| Sample pad | C083 Cellulose fiber | Millipore | CFSP001700 | 9x3 | LFIA |
| Sample pad | Glass fiber | Millipore | GFCP103000 | 13.8x12.5 | Duplex |
| Absorbent pad | Whatman CF5 | Cytiva | 8115-2250 | 30x3^1^,12x3^2^ | Dipstick^1^, LFIA^2^ |
| Backing card | Grafix Adhesive-Backed Film | Blick Arts | 55525-1021 | 58x3^1^, 50x3^2^, 13.8x58.5^3^ | Dipstick^1^, LFIA^2^, Duplex^3^ |
| Commercial Assay | Universal Nucleic Acid Detection Strip | Ustar Biotechnologies | U40009 | 50x4.2 | Duplex |
